# Job vacancy at the European Centre for Disease Prevention and Control

**DOI:** 10.2807/1560-7917.ES.2017.22.25.30554

**Published:** 2017-06-22

**Authors:** Eurosurveillance Editorial team

**Affiliations:** 1 European Centre for Disease Prevention and Control (ECDC), Stockholm, Sweden

The European Centre for Disease Prevention and Control (ECDC) invites applications for the following position:

Expert Outbreak Response

The deadline for the application is 16 August 2017. For more information, visit the ECDC website:

http://ecdc.europa.eu/en/aboutus/jobs/Pages/JobOpportunities.aspx

